# Unlocking Infertility: Enhancing Pregnancy Rates With Personalized Embryo Transfers Using Optimal Time for Endometrial Receptivity Analysis in Recurrent Implantation Failure Patients Undergoing In Vitro Fertilization

**DOI:** 10.7759/cureus.54940

**Published:** 2024-02-26

**Authors:** Hiren Gajjar, Jwal Banker, Shiva Murarka, Parth Shah, Nidhi Shah, Lakshmi Bhaskaran

**Affiliations:** 1 Reproductive Genetics, Neuberg Center for Genomic Medicine, Ahmedabad, IND; 2 Gynecology, Nova Pulse IVF Center, Ahmedabad, IND; 3 Department of Pathology and Lab Medicine, Dartmouth Hitchcock Medical Center, Lebanon, USA; 4 Department of Biotechnology and Microbiology, Kadi Sarva Vishwavidyalaya, Gandhinagar, IND

**Keywords:** opera, recurrent implantation failure, personalized embryo transfer, pre-implantation genetic testing, window of implantation

## Abstract

Background

Infertility remains a significant global challenge, and recurrent implantation failure (RIF) poses a considerable concern in assisted reproductive technology. Understanding the factors contributing to implantation failure is essential for developing accurate diagnostic tools and treatment strategies. Endometrial receptivity (ER) during the window of implantation is crucial for successful embryo implantation in in vitro fertilization (IVF) procedures. Molecular-based endometrial receptivity analysis and next-generation sequencing provide insights into ER, but there is a lack of research on these in the Indian population, particularly in patients with RIF. This retrospective cohort study evaluates the effectiveness of Optimal Timing for Endometrial Receptivity Analysis (OpERA)-guided personalized embryo transfer (pET) in Indian patients with a history of RIF.

Methodology

The study includes 158 female patients with a history of failed embryo transfers who underwent OpERA testing before frozen embryo transfer. Patients were categorized based on the number of previous failed transfers. OpERA outcomes were assessed, and clinical outcomes were compared between groups undergoing preimplantation genetic testing for aneuploidy (PGT-A) with and without OpERA. Endometrial preparation involved hormone replacement therapy, and OpERA testing was performed at the Neuberg Centre for Genomic Medicine using RNA extraction, cDNA conversion, and sequencing.

Results

OpERA outcomes showed no significant differences in receptive rates among patient groups. Group 3, with three or more failed transfers, exhibited significantly higher biochemical pregnancy rates (BPRs), clinical pregnancy rates (CPRs), and abortion rates (ARs) compared to Groups 1 and 2. OpERA with PGT-A showed significantly higher BPR, implantation rate, CPR, and lower AR compared to OpERA without PGT-A.

Conclusions

OpERA-guided pET, especially with PGT-A, demonstrated improved pregnancy outcomes, particularly in patients with a history of RIF. The study emphasizes the importance of OpERA in determining optimal transfer timing, moving beyond the traditional reliance on embryo quality alone. OpERA presents promise in predicting pregnancy outcomes for Indian patients with previous IVF failures. The integration of OpERA and PGT-A represents a significant advancement in personalized reproductive medicine, offering new hope for individuals grappling with infertility complexities.

## Introduction

Infertility remains a major obstacle in the field of reproductive medicine, affecting numerous couples worldwide [1]. Recurrent implantation failure (RIF) continues to be a considerable concern despite notable progress in assisted reproductive technology (ART). This denotes the inability to attain clinical pregnancy despite the transfer of multiple high-quality embryos [2]. To effectively deal with this persistent problem, having a comprehensive understanding of the factors that lead to implantation failure and promoting the development of precise tests and treatment approaches is of utmost importance.

Embryo implantation is an intricate procedure that relies on healthy embryo-receptive interaction with a specific focus on the timeframe known as the window of implantation (WOI) [3]. It is commonly known that endometrial receptivity (ER) is important during this crucial period because it determines the endometrium’s capacity to accept and sustain a viable embryo, which, in turn, affects the outcome of in vitro fertilization (IVF) procedures [4]. Molecular signatures and gene expression profiles associated with receptive (R) endometrium have been provided by molecular-based endometrial receptivity (ERA) and next-generation sequencing (NGS) techniques, which have revolutionized the ability to assess and predict ER [5].

However, there is a significant lack of research on the evaluation of ER and personalized embryo transfer (pET), specifically in the Indian population. Investigation into the feasibility of these approaches is warranted in India due to the country’s distinct demographic features, particularly in patients with RIF. The primary objective of this retrospective cohort study was to assess the effectiveness of optimal time for endometrial receptivity analysis (OpERA)-guided pET in improving reproductive outcomes for Indian patients who had experienced multiple ET failures. Additionally, the study aimed to evaluate the potential benefits of combining OpERA-guided pET with preimplantation genetic testing for aneuploidy (PGT-A) in this population.

## Materials and methods

Study design

A total of 158 female patients undergoing comprehensive IVF evaluations, with no underlying reasons for multiple failed embryo transfer (ET) cycles, were assessed across various factors, including male factor, combined male and female factor, and unexplained infertility. All patients with a history of previous failed ET underwent OpERA testing followed by frozen embryo transfer (FET) with hormone replacement therapy (HRT). The patients were then categorized into three distinct groups based on the number of previous failed ET cycles, namely, Group 1 (zero or one prior failed ET), Group 2 (two prior failed ETs), and Group 3 (≥three prior failed ETs). Patients were excluded if they had untreated hydrosalpinx, submucosal polyps, cavity-compressing fibroids, or any untreated pathology that could potentially affect the implantation. A tertiary infertility clinic, Nova IVF Fertility, Ahmedabad, from July 2022 to March 2023, served as the study site. The research was conducted in strict compliance with ethical standards, following the guidelines laid out by the International Council on Harmonization’s Good Clinical Practice Guidelines (E6, 1996) and the Declaration of Helsinki (Brazil, 2013), among other applicable regulatory requirements. To assess ER, the OpERA test was performed only after all patients in the cohort had provided written informed consent.

The primary outcome measures in this study focused on assessing the effectiveness of OpERA, with particular emphasis on the biochemical pregnancy rate (BPR), clinical pregnancy rate (CPR), implantation rate (IR), and abortion rate (AR). Furthermore, a comparative statistical analysis was conducted to evaluate the clinical outcomes between groups that underwent OpERA with and without PGT-A.

Endometrium preparation and collection

All patients underwent HRT following a standardized FET protocol. On cycle day two, a transvaginal ultrasound was performed to rule out any cysts or pathologies. Endometrial preparation commenced on day two, involving the administration of 8 mg estradiol hemihydrate in two divided doses. Serial ultrasounds were conducted until the endometrial thickness reached a threshold of >7 mm. Serum progesterone levels were monitored, and individuals with levels below 0.5 ng/mL received progesterone supplementation. Estradiol administration continued, and progesterone medication was given for five days as per the blastocyst transfer protocol. After five days, endometrial sampling was performed using a pipelle, and endometrial tissue was collected and stored for OpERA testing.

OpERA test and window of implantation recommendations

The collected endometrial samples were sent to the Neuberg Center for Genomic Medicine (NCGM) for the OpERA test. The OpERA test involved RNA extraction from the endometrial samples, followed by the conversion of RNA into complementary DNA (cDNA) through reverse transcription. Subsequently, cDNA was amplified and sequenced using the advanced Illumina Novaseq 6000 platform. RNA expression levels were evaluated using the fragments per kilobase of transcript per million mapped reads. ER conditions were assessed using a machine-learning model based on the predicted gene assortment from the NCGM. The OpERA test provided outcomes indicating the endometrium’s receptive (R) or non-receptive (NR) state. NR test results were further categorized as pre-receptive or post-receptive, signifying a potentially displaced WOI. Based on these OpERA results, embryos were transferred during the maximum permissible WOI. If OpERA results indicated a pre-receptive phase, it was recommended to delay the transfer; conversely, if the OpERA results indicated a post-receptive phase, it was advised to advance the transfer timing.

Embryo chromosomal analysis before implantation

Embryo screening was performed using NGS at NCGM in Ahmedabad. PGT-A is used to ascertain the genetic condition of the embryo, specifically whether it is euploid, aneuploid, or mosaic. Euploid embryos had two copies of the chromosome, monosomy had one, and trisomy had three, according to biopsy, NGS screening, and analysis. Values falling within the range of one to two or two to three were categorized as a mosaic. Aneuploidy was defined as more than 80% of mosaicism, euploidy as less than 20%, and mosaicity as 20% to 80%. Following ERA testing, patients underwent pET after embryo screening, and clinical pregnancy was verified by evaluating the level of the β-subunit of human chorionic gonadotropin 15 days after ET.

Statistical analysis

This study was to attain at least equivalence, guided by a statistical power analysis that set a sample size of 158, ensuring an 80% confidence level with a 5% margin of error for robust detection of meaningful differences or equivalence among the groups. The statistical analysis involved a three-way analysis of variance (ANOVA) and Student’s t-test. A significance level of p < 0.05 was applied to assess the statistical significance of quantitative continuous and categorical variables using SPSS software (IBM Corporation, Armonk, NY, USA). Demographic variables were summarized using means and percentages. Multiple pairwise comparisons of means from the ANOVA data were conducted using Tukey’s honestly significant difference (HSD) method. P-values were utilized to determine the presence or absence of substantial differences between sample means.

## Results

Patient characteristics and OpERA test results

In this study, 158 patients with varying numbers of prior failed ET cycles were analyzed and categorized into the following three groups based on their ET history. Group 1 included 38 patients with ≤one prior failed ET, Group 2 comprised 67 patients with two prior failed ET cycles, and Group 3 contained 53 patients with ≥three prior failed ET cycles. Blastocyst quality was assessed using Gardner’s scoring system, with a specific focus on defining a good-quality blastocyst as one with a Gardner’s score of 4BB or higher. Notably, blastocyst quality was found to be comparable across all three groups.

The mean age in each group was 35.2 ± 4.5 years, 33.3 ± 4.5 years, and 33.9 ± 4.5 years, respectively, with no statistically significant age differences among the groups (p = 0.110). Similarly, the mean body mass index (BMI) was 27.0 ± 5.5 kg/m^2^, 26.9 ± 5.4 kg/m^2^, and 26.9 ± 5.5 kg/m^2^ for patients in the respective groups, showing no significant BMI variations (p = 0.994). Endometrial thickness displayed mean values of 8.1 ± 0.9 mm, 8.0 ± 1.0 mm, and 7.9 ± 1.1 mm for the three groups, with no significant differences observed (p = 0.327). The mean total number of embryos transferred was 1.7 ± 0.6, 1.6 ± 0.5, and 1.6 ± 0.5 for patients in the respective groups, with a non-significant difference (p = 0.052) (Table 1).

**Table 1:** Characteristics of patients selected for the study. A indicates the three-way analysis of variance statistical analysis. SD: standard deviation; OpERA: optimal time for endometrial receptivity analysis; ET: embryo transfer

	≤1 prior failed ET	2 prior failed ET	≥3 prior failed ET	P-value
Number of patients	38	67	53	
Age (years ± SD)	35.2 ± 4.5	33.3 ± 4.5	33.9 ± 4.5	0.110^A^
Body mass index (BMI) (kg/m^2^)	27.0 ± 5.5	26.9 ± 5.4	26.9 ± 5.5	0.994^A^
Endometrium thickness (mm)	8.1 ± 0.9	8.0 ± 1.0	7.9 ± 1.1	0.327^A^
Total number of embryos transferred	1.7 ± 0.6	1.6 ± 0.5	1.6 ± 0.5	0.052^A^
Reason for infertility				
Male factor	7/38	11/67	10/53	
Combined male and female factor	2/38	4/67	4/53	
Unexplained	29/38	52/67	39/53	
OpERA results				
Receptive (R)	21/38 (55%)	31/67 (46%)	27/53 (51%)	0.670^A^
Pre-receptive	13/38 (34%)	33/67 (49%)	23/53 (43%)	0.331^A^
Post-receptive	4/38 (11%)	3/67 (4%)	3/53 (6%)	0.464^A^

OpERA test results showed that 55% of patients in Group 1, 46% in Group 2, and 51% in Group 3 were classified as receptive (R). The percentages of pre-receptive and post-receptive cases exhibited no significant differences among the groups (p = 0.331 and p = 0.464, respectively). These results underscore the lack of significant variation in patient characteristics and OpERA test outcomes among the distinct patient groups (Table 1).

Clinical outcomes of OpERA in three patient groups

In this study, the clinical outcomes of the OpERA test were assessed in three distinct patient groups. The clinical outcomes are reported as percentages of patients achieving BPR, CPR, IR, and AR following OpERA. In Group 1, the BPR following OpERA was 57.9% compared to 50.7% in Group 2 and a notably higher rate of 81.1% in Group 3 (p = 0.0020). The CPR following OpERA was 50% for patients in Group 1, 43% for those in Group 2, and 79% for patients in Group 3, showing a statistically significant difference (p = 0.0002). The IRs following OpERA were 55.9% (19/34) for ≤one prior failed ET, 57.1% (28/49) for two prior failed ET cycles, and 55.4% (41/74) for ≥three prior failed ET cycles. A p-value of 0.9822 was obtained for the comparison of IRs among the three groups, signifying the absence of significant differences. The AR following OpERA was 42% for patients in Group 1, 49% for Group 2, and 19% for Group 3, indicating a significant variation among the groups (p = 0.0020) (Table 2).

**Table 2:** Clinical outcomes of OpERA in three different groups. A indicates the three-way analysis of variance statistical analysis; * indicates the statistically significant value (p < 0.05). OpERA: optimal time for endometrial receptivity analysis; BPR: biochemical pregnancy rate; CPR: clinical pregnancy rate; IR: implantation rate; AR: abortion rate; ET: embryo transfer

	≤1 prior failed ET	2 prior failed ET	≥3 prior failed ET	P-value
Number of patients	38	67	53	
BPR following OpERA outcomes (%)	22/38 (57.9%)	34/67 (50.7%)	43/53 (81.1%)	0.0020*^,A^
CPR following OpERA outcomes (%)	19/38 (50%)	28/65 (43%)	41/52 (79%)	0.0002*^,A^
IR following OpERA outcomes (%)	19/34 (55.9%)	28/49 (57.1%)	41/74 (55.4%)	0.9822^A^
AR following OpERA outcomes (%)	16/38 (42%)	33/67 (49%)	10/53 (19%)	0.0020*^,A^

Pregnancy outcomes in OpERA patients with and without PGT-A

The impact of PGT-A on pregnancy outcomes among patients who underwent OpERA was investigated. In the study, 44 (35%) patients received OpERA with PGT-A, while 82 (65%) patients had OpERA without PGT-A. The analysis revealed no statistically significant difference in the number of prior failed ET cycles between the OpERA with PGT-A group (mean ± SD = 2.32 ± 1.03) and the OpERA without PGT-A group (mean ± SD = 2.46 ± 1.03) (p = 0.4684). In the OpERA with PGT-A group, the BPR was 70.5%, whereas the OpERA without PGT-A group had a BPR of 54.9% (p = 0.0448). Significant differences were also observed in the IR, with the OpERA with PGT-A group achieving a substantially higher rate of 52.6% compared to the OpERA without the PGT-A group’s rate of 27.2% (p = 0.0001). Similarly, the CPR showed significant disparities, with 62.8% for the OpERA with PGT-A group and 46.9% for the OpERA without PGT-A group (p = 0.0467). However, AR exhibited a different pattern, with the OpERA with PGT-A group at 29.6%, which was lower compared to the OpERA without PGT-A group’s rate of 46.3% (p = 0.0339) (Table 3).

**Table 3:** Pregnancy outcome in PGT-A patients and non-PGT-A patients with OpERA results. A indicates the t-test statistical analysis; * indicates the statistically significant value (p < 0.05). OpERA: optimal time for endometrial receptivity analysis; ET: embryo transfer; BPR: biochemical pregnancy rate; CPR: clinical pregnancy rate; AR: abortion rate; IR: implantation rate; PGT-A: pre-implantation genetic testing for aneuploidy

	OpERA with PGT-A	OpERA without PGT-A	P-value
Number of patients	44 (35%)	82 (65%)	
Number of prior failed ET	2.32 ± 1.03	2.46 ± 1.03	0.4684^A^
BPR (%)	31/44 (70.5%)	45/82 (54.9%)	0.0448*^,A^
IR (%)	40/76 (52.6%)	40/147 (27.2%)	0.0001*^, A^
CPR (%)	27/43 (62.8%)	38/81 (46.9%)	0.0467*^, A^
AR (%)	13/44 (29.6%)	38/82 (46.3%)	0.0339*^, A^

## Discussion

RIF remains a significant challenge in ART and is characterized by the absence of pregnancy after three or more IVF attempts using high-quality embryos [6]. This study specifically focused on cases within Group 3, emphasizing patients who had experienced three or more unsuccessful ET cycles. It is important to highlight that, despite transferring euploid embryos, achieving a 100% success rate in IVF is not assured, as various factors beyond embryonic abnormalities influence the outcome. Although embryo quality is undoubtedly crucial, the receptive (R) state of the endometrium also plays a significant role in successful implantation [7].

This study aimed to evaluate ER using the OpERA tool and explore the potential benefits of integrating PGT-A with OpERA to improve pregnancy outcomes. Previous studies have consistently emphasized the complex and diverse characteristics of RIF, encompassing both embryonic and endometrial factors [8,9]. To effectively assess ER, achieving the receptive (R) stage is essential for successful embryo implantation, especially during WOI. Accurately predicting the timing of WOI and performing accurate ET within this crucial timeframe has the potential to significantly improve pregnancy rates and increase the chances of successful pregnancies.

Numerous methods have been used to determine the WOI, such as ultrasound measurement, examination of endometrial pinopodes, and examination of serum molecules. Nevertheless, these techniques have constraints in precisely ascertaining ER for clinical applications [10]. Molecular-based ER assessment for accurate ET has demonstrated encouraging results, exhibiting elevated pregnancy rates for the receptive (R) endometrium in contrast to non-receptive (NR) instances [11]. A multicenter clinical study found that ERA-guided ET resulted in notable improvements in ongoing pregnancy rates and IRs among patients with RIF [12]. A retrospective study conducted at an Indian infertility clinic found that a significant majority of patients with unexplained RIF had receptive (R) endometrium [7].

A decreased proportion of receptive (R) endometrium was identified using OpERA analysis in our study compared with the results reported in earlier studies. A retrospective analysis of 97 patients who had undergone multiple unsuccessful implantations revealed that ERA testing successfully identified the receptive (R) endometrium in approximately 48.5% of the cases [13]. The discrepancies in the rates of receptive (R) endometrium observed in various studies can be linked to discrepancies in patient demographics, geographical variables, treatment methods, and evaluation methods, such as collection timing and parameters for determining receptivity. Conditions related to the female reproductive system, including diseases in the fallopian tubes, ovarian issues, and endometriosis, can affect the endometrium’s ability to receive and support pregnancy [14,15]. Furthermore, the utilization of HRT cycles may have an impact on progesterone administration, which could potentially influence receptivity [16].

A recent randomized controlled trial revealed that ERA-guided precision ET resulted in enhanced rates of pregnancy, implantation, and cumulative live births in patients undergoing IVF compared to both frozen and fresh ET methods. Nevertheless, this study did not include patients with RIF due to inconclusive findings from previous studies [12,11,17,18]. Our study revealed noteworthy enhancements in Group 3, comprising individuals diagnosed with RIF. Group 3 exhibited higher BPR and CPR than Groups 1 and 2, indicating that OpERA-guided ET can improve outcomes.

These findings emphasize the significance of OpERA in determining the most favorable timeframe for ET, especially in difficult cases, such as RIF. This study enhances the current body of research by providing valuable insights into the potential of OpERA as a tool for guiding pET in patients who have experienced multiple unsuccessful implantations. This approach deviates from traditional methods that frequently depend solely on embryo quality, introducing a more sophisticated comprehension of the significance of ER.

Contrary to our results, PGT-A demonstrated benefits for moderate recurrent implantation failure, with increased implantation and ongoing pregnancy rates, while no significant improvements were observed in severe cases; ERA did not show clinical utility in either group [19]. Similarly, a recent study found that precision ET guided by ERA did not yield any clinical advantages in patients who had previously experienced implantation failure [14]. The discrepancies in research findings can be ascribed to differences in patient attributes, treatment procedures, and other variables that can affect the effectiveness of precise ET guided by ERA. They emphasized the significance of conducting additional research to gain a deeper understanding of the efficacy and consequences of precision ET.

Recent research has provided insights into the significance of substandard embryo quality, as well as the receptiveness of the endometrium, in cases of RIF [20]. Our study employed PGT-A to ascertain the genetic condition of the embryos, enabling a more accurate assessment of their role in RIF. NGS-based PGT-A is considered a valuable improvement to the current methods for addressing RIF.

The results of our study indicated that the OpERA group, which underwent PGT-A, showed significantly higher BPR and CPR than the non-PGT-A cohort. More precisely, within the OpERA group, there was a notable enhancement in BPR and CPR compared to the non-PGT-A cohort. Genetically screened embryo selection is likely to enhance implantation success and reduce the occurrence of AR failures. Furthermore, the PGT-A group exhibited superior rates of ongoing pregnancy and implantation in patients with RIF, suggesting its potential to enhance reproductive success. These findings provide valuable information to clinicians and patients.

This study has several limitations, one of which is its retrospective nature. This means that bias may have been introduced when compared to prospective clinical trials. Additionally, the absence of control samples is noted, although initial levels were considered for the validation test. Furthermore, the small sample size may have hindered the application of our findings to a broader population. The utilization of self-reported instruments for data collection introduces the potential for reporting inaccuracies or prejudice, and the design permitted a time interval of up to six months between the measurement of biochemical parameters and data collection. In addition, the lack of data on live births is a limitation of this study. However, detailed records of clinical pregnancies and early pregnancy losses offer reliable information on overall clinical outcomes, with very few instances of late pregnancy loss. This finding strengthens the credibility of our findings.

## Conclusions

OpERA, a molecular-based test for ER, shows promise for predicting pregnancy outcomes in Indian patients who have previously experienced IVF failure. Utilizing OpERA-guided ET, especially when combined with PGT-A, leads to higher pregnancy rates. Synchronizing ET with OpERA showed the potential for enhanced success. OpERA shows the potential for addressing RIF in ART; nevertheless, further research and extensive trials are required to assess its impact on live birth rates. The integration of OpERA and PGT-A signifies significant advancement in personalized and precision-oriented reproductive medicine. This development signifies a fundamental change in how difficult cases such as RIF are approached. The practical significance of our findings highlights the capacity of OpERA to serve as a valuable tool for medical practitioners, providing fresh optimism for individuals confronting the intricacies of infertility.
